# 3D-Printed Modular Microfluidic Device Enabling Preconcentrating Bacteria and Purifying Bacterial DNA in Blood for Improving the Sensitivity of Molecular Diagnostics

**DOI:** 10.3390/s20041202

**Published:** 2020-02-21

**Authors:** Abdurhaman Teyib Abafogi, Jaewon Kim, Jinyeop Lee, Merem Omer Mohammed, Danny van Noort, Sungsu Park

**Affiliations:** 1 School of Mechanical Engineering, Sungkyunkwan University, Suwon 16419, Korea; ab18aa@gmail.com (A.T.A.); jaewon1394@gmail.com (J.K.); softmemsljy@naver.com (J.L.); 2 Department of Biomedical Engineering, Sungkyunkwan University, Suwon 16419, Korea; meremomer3@gmail.com; 3 Division of Biotechnology, IFM, Linkoping University, 58183 Linkoping, Sweden; drr.dvn@gmail.com; 4 Chair of Micro Process Engineering and Technology (COMPETE), University of Ljubljana, 1000 Ljubljana, Slovenia; 5 Centro de Investigación en Bioingeniería -BIO, Universidad de Ingenieria y Tecnologia—UTEC, Barranco 15036, Peru; 6 Biomedical Institute for Convergence at SKKU (BICS), Sungkyunkwan University, Suwon 16419, Korea

**Keywords:** molecular diagnostics, microfluidic device, bacterial preconcentration, DNA purification, pathogen

## Abstract

Molecular diagnostics for sepsis is still a challenge due to the presence of compounds that interfere with gene amplification and bacteria at concentrations lower than the limit of detection (LOD). Here, we report on the development of a 3D printed modular microfluidic device (3DpmμFD) that preconcentrates bacteria of interest in whole blood and purifies their genomic DNA (gDNA). It is composed of a W-shaped microchannel and a conical microchamber. Bacteria of interest are magnetically captured from blood in the device with antibody conjugated magnetic nanoparticles (Ab-MNPs) at 5 mL/min in the W-shaped microchannel, while purified gDNA of the preconcentrated bacteria is obtained with magnetic silica beads (MSBs) at 2 mL/min in the conical microchamber. The conical microchamber was designed to be connected to the microchannel after the capturing process using a 3D-printed rotary valve to minimize the exposure of the MSBs to interfering compounds in blood. The pretreatment process of spiked blood (2.5 mL) can be effectively completed within about 50 min. With the 3DpmμFD, the LOD for the target microorganism *Escherichia coli* O157:H7 measured by both polymerase chain reaction (PCR) with electrophoresis and quantitative PCR was 10 colony forming unit (CFU) per mL of whole blood. The results suggest that our method lowers the LOD of molecular diagnostics for pathogens in blood by providing bacterial gDNA at high purity and concentration.

## 1. Introduction

Sepsis is a life-threatening immune response caused by a bacterial infection in blood [1,2,3], causing approximately 6 million deaths worldwide each year [4]. Early detection of sepsis is necessary to provide appropriate treatment and reduce mortality [5]. Molecular diagnostics based on gene amplification is rapid and accurate for detection of microorganisms [6,7]. However, its use in blood without extraction and purification of bacterial target genomic DNA (gDNA) often fails to obtain sensitive results [8] because blood contains indigenous compounds that interfere with gene amplification [6,8,9,10,11].

gDNA purification methods can also improve the limit of detection (LOD) of molecular diagnostics for sepsis by providing purified gDNA at high concentrations [11] for use with polymerase chain reaction (PCR) and quantitative PCR (qPCR). Existing purification methods include alkaline extraction [12], gradient centrifugation [13], and magnetic silica bead (MSB)-based gDNA extraction [14]. Among these methods, MSB-based gDNA extraction has been frequently used because of its simplicity [15]. Thus, gDNA extraction steps are prerequisites for molecular diagnostics for microorganisms in blood [10]. Commercialized gDNA extraction kits have been widely used to extract and purify bacterial gDNA from infected blood [13]. However, most of the commercialized kits handle hundreds of microliters of sample and obtain hundreds of microliters of bacterial gDNA, therefore the preconcentration effect is low. Out of the hundreds of microliters obtained by these kits most of it is not used for amplification because both PCR and qPCR typically use only several microliters of purified gDNA for amplification [16]. Thus, the improvement of LOD achieved with these kits is not substantial. Consequently, there is an urgent need for devices that preconcentrate bacteria in a large volume of blood and then obtain highly concentrated bacterial gDNA in a small volume [14].

Various methods, including immunomagnetic separation (IMS), have been developed to separate and preconcentrate bacteria of interest from compounds that interfere with gene amplification [17,18]. IMS has been often used in microfluidic devices (μFDs) made of polydimethylsiloxane (PDMS). However, these μFDs can handle only a small volume of sample [19] because they consist of thin microchannels. Molds for the μFDs are usually made by photolithography, which is known to be not suitable for fabricating round structures such as spiral and circular channels [20,21]. Unlike photolithography, round structures as well as high aspect ratio structures can be fabricated using 3D printing [14,21,22]. Therefore, μFDs fabricated by 3D printing are suitable for processing a high volume of sample and their structures can be flexibly designed.

Recently, it was reported that a 3D-printed microfluidic (3DpmμFD) with a helical microchannel for IMS of pathogenic bacteria in food achieved flow rates of up to 5 mL/min [23]. More recently, we have developed a 3DpmμFD that performs both IMS preconcentration and MSB-based gDNA extraction in a trapezoidal microchamber [14]. However, this device could not be used directly with whole blood because the MSBs were contaminated with compounds that interfere with gDNA amplification, requiring a 10-fold dilution of blood samples to obtain purified gDNA. The result suggests that the exposure of MSBs to blood should be minimized to protect the silica surfaces from being fouled by blood.

**Figure 1:**
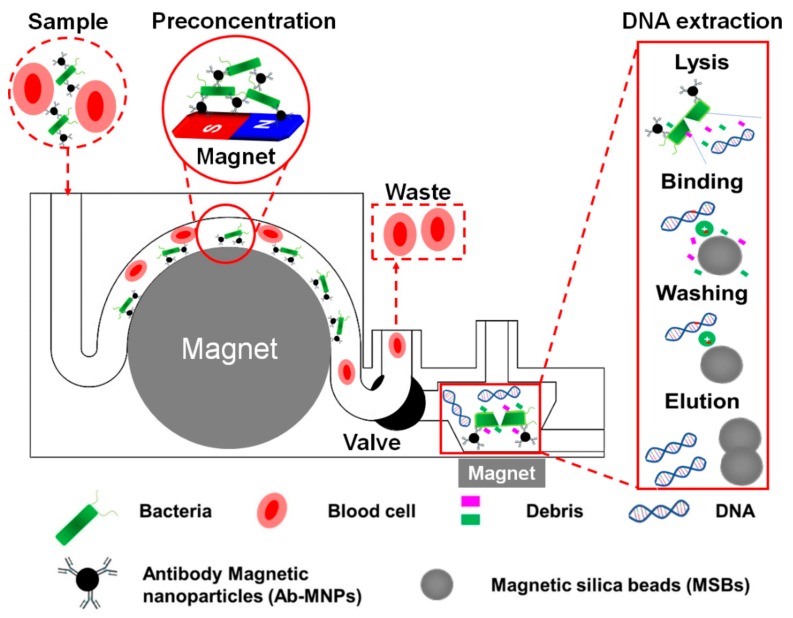
Schematic describing preconcentrating bacteria and purifying bacterial genomic DNA in blood using 3DpmμFD. *E. coli* O157:H7 in blood was first preconcentrated with magnetic nanoparticles (MNPs) conjugated with antibody (Ab) specific for the serotype in the microchannel using a permanent magnet (preconcentration step). Then, only bacteria captured with Ab-MNPs were transported from the microchannel module into a conical microchamber where the cells were lysed, and bacterial gDNA was adsorbed onto the silica surface of MSBs by chaotropic salts (DNA extraction step). Through these steps, preconcentrated bacterial gDNA can be obtained from the blood sample using 3DpmμFD.

In this study, we report on the development of a 3D printed modular microfluidic device (3DpmμFD) that is assembled with a microchannel module for bacterial preconcentration and a conical microchamber module for DNA purification. To minimize the fouling of MSBs with blood, bacteria of target in blood was first preconcentrated with magnetic nanoparticles (MNPs) conjugated with antibody (Ab) specific for the target bacteria in the microchannel module using a permanent magnet (Figure 1). Then, only bacteria captured with Ab-MNPs were transported from the microchannel module into a conical microchamber where the cells were ruptured, and genomic gDNA was adsorbed onto the silica surface of MSBs by chaotropic salts (Figure 1). To demonstrate the feasibility of applying the 3DpmμFD to blood, blood was spiked with *Escherichia coli* O157:H7, and Ab-MNPs were prepared with antibody specific to the serotype. Using the 3DpmμFD, as low as 10 *E. coli* O157:H7 colony forming units (CFUs) per mL in 2.5 mL of blood were detectable by both PCR and qPCR.

## 2. Materials and Methods

### 2.1. Reagents

Sodium tetraborate, 4-morpholineethanesulfonic acid (MES), glutaraldehyde, and sodium cyanoborohydride were purchased from Sigma-Aldrich. Bovine serum albumin (BSA) and phosphate-buffered saline (PBS, pH 7.4) were purchased from Gibco (Grand Island, NY, USA).

### 2.2. Bacterial Culture

The bacterial strains used in this study include *E. coli* O157: H7 (ATCC 43894), *Staphylococcus aureus* (ATCC 27213), and *Salmonella enteritidis* (ATCC 13076) from American Type Culture Collection (ATCC, Bethesda, MD, USA). A single colony on an agar plate seeded with each strain was transferred and inoculated into 5 mL of Luria–Bertani (LB) broth (Becton, Dickinson, and Company, Franklin Lakes, NJ, USA). The culture was then incubated overnight at 37 °C and 200 rpm. Finally, the overnight culture was diluted 100-fold with fresh LB broth and incubated at the same conditions until the optical density (OD) at 600 nm reached 1.

### 2.3. Synthesis of Ab-MNPs

50 μg of affinity-purified anti-*E. coli* O157:H7 antibody from KPL (Gaithersburg, MD, USA) was conjugated to 1 mg of amine-functionalized MNPs (100 nm diameter) from Chemicell Co. (Berlin, Germany) as reported previously [14].

### 2.4. D Printing

**Figure 2:**
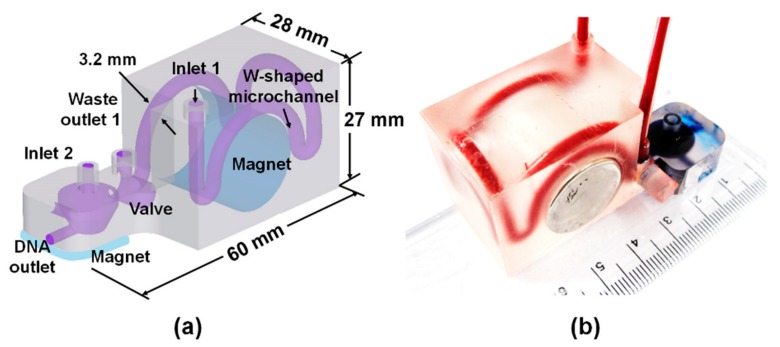
3DpmμFD for preconcentrating bacteria and purifying genomic bacterial DNA in blood. (**a**) Design and operation of the microchannel module for magnetic preconcentration of bacteria of interest. (**b**) Photographic image of the W-shaped microchannel and conical microchamber.

The student edition of Inventor® professional (Autodesk Inc., Seoul, Korea) was used to design the 3DpmμFD model. For bacterial preconcentration, the W-shaped microchannel (3.2 × 26.3 mm: width × length) was designed (Figure 2a). For gDNA purification, a conical microchamber (13 × 7 × 7 mm: diameter × length × round bottom diameter) and a valve (6 × 10 mm) were separately designed and manually assembled with the W-shaped microchannel (Figure 2a). Helical shaped microfluidic device with one revolution (3.2 × 72 mm: width × length), two revolutions (3.2 × 140 mm: width × length), and three revolutions (3.2 × 210 mm: width × length) was designed and fabricated. The 3D model of each device was then sliced into 100-µm-thick layers in the z-axis direction. Each layer was printed using a digital light processing (DLP) printer (IM-96, Carima Co., Seoul, Korea) by exposing the photosensitive acrylic resin (Carima Co.) to UV and developing it layer by layer. The residual resin in the printout was then removed by washing with 70% ethanol. The strength of the printout was enhanced by UV treatment for 10 min. The W-shaped microchannel, conical microchamber, and valve were manually assembled to complete the 3DpmμFD (Figure 2b).

### 2.5. Estimation of the Capturing Efficiency Using Computational Fluid Dynamics (CFD) Analysis 

CFD simulation was performed using COMSOL Multiphysics^®^ software (ver. 5.1) to analyze the behavior of bacterium-Ab-MNP complexes in both W-shaped (Figure 2) and helical microchannels (Figure S1). To calculate the bacterial capturing efficiency of the W-shaped microchannel, one hundred bacterium-Ab-MNP complexes were injected from the inlet into the microchannel at a flow rate of 2 mL/min. Average magnetic field applied to the W-shaped and helical microchannels was 528 mT and 115 mT, respectively. Bacterial capturing efficiency was estimated by dividing the number of captured complexes in the channel one hundred bacterium-Ab-MNP complexes.

### 2.6. Bacterial Capture by 3DpmμFD

The use of blood was approved by the Institutional Review Board (IRB) of Sungkyunkwan University (SKKU) (approval number SKKU 2017-11-006). Blood from Innovative Research, Inc. (Novi, MI, USA) was treated with 0.1% K2 ethylenediaminetetraacetic acid (EDTA). Ab-MNPs (200 μL, final concentration, 10^11^ particles/mL) were mixed with 2.5 mL of blood that had been spiked with either *E. coli* O157:H7 or *S. aureus* or *S. enteritidis* (final concentration, 10^5^ CFU/mL). This mixture was incubated in a glass beaker at 37 °C and 200 rpm for 20 min. A permanent magnet (diameter: 20 mm, height: 20 mm, magnetic flux density: 560 MT) was then plugged into the hole of the W-shaped channel (Figure 2a). Then, the mixture was injected into the inlet 1 of the 3DpmμFD at various flow rates (2–20 mL/min) with a syringe pump (Harvard Apparatus, Boston, MA, USA) while closing the channel connected to the conical chamber using the valve. The preconcentration steps took about 1 min at 2 mL/min. Eluents collected from the waste outlet 1 during the preconcentration were collected and inoculated in agar plates for the standard colony counting [24]. The colony numbers were used to calculate the number of uncaptured bacterial cells during the preconcentration [22]. The following equation was used to calculate the capturing efficiency of the devices [22].

(1) $$Capturing\;efficency\;\left(\%\right)=\left(\frac{N_{t}-N_{u}}{N_{t}}\right)100\%,$$

where N_t_ is the number of the total bacterial cells in the sample, and N_u_ is the number of uncaptured bacterial cells in the sample.

### 2.7. Bacterial gDNA Purification on 3DpmμFD with a Conical Microchamber

For DNA purification, the permanent magnet was first removed from the hole of the W-shaped channel and 100 µL of PBS were then injected at 2 mL/min into the W-shaped channel to transfer preconcentrated bacteria with Ab-MNPs from the channel to the conical microchamber while opening the channel connected the chamber using the valve.

Once preconcentrated bacteria with MNPs were located into the chamber, bacteria lysis, DNA binding, washing, and elution steps were performed in a sequential manner using the reagents of MagListo™ 5M Genomic DNA extraction kit (Bioneer Co.) and the manufacturer’s protocol with some modifications as follow. In brief, 100 µL of lysis buffer (MagListo™ 5M Genomic DNA extraction kit) were added into the chamber through the MSB inlet (Figure 2a) using a pipette and incubated at 60 °C for 10 min on a hotplate (Daihan Scientific Co., Wonju, Korea) until bacteria were lysed while both DNA and waste two outlets were closed with metal plugs. After the lysis step, 100 µL of a mixture of binding buffer (MagListo™ 5M Genomic DNA extraction kit) with MSBs (500 nm in diameter, MagListo™ 5M Genomic DNA extraction kit) at 10^11^ particles/mL (final conc.) were added into the chamber through the MSB inlet using a pipette (Figure 2a). Absolute ethanol (200 µL) were additionally added to the chamber through the same inlet. Bacterial cell debris was removed from the chamber using gravity-driven flow by opening the waste outlet 2 while placing a rectangular permanent magnet under the microchamber. Then, 300 µL of wash buffer (MagListo™ 5M Genomic DNA extraction kit) were added to the chamber through the MSB inlet while closing the waste outlet 2. Outlet 2 was opened again to remove residual cell debris from the chamber while placing the permanent magnet under the microchamber. Finally, 50 µL of elution buffer (MagListo™ 5M Genomic DNA extraction kit) were added to the chamber while closing the waste outlet 2 and opening the DNA outlet. In this way, purified gDNA from the DNA outlet was pooled to about 50 uL in a 1.5 mL tube and used for analysis of DNA purity and following PCR and qPCR. Bacterial gDNA extraction step took about 30 min.

### 2.8. Analysis of DNA Purity and Yield

After gDNA extraction using either 3DpmμFD or the three commercial kits (MagListoTM 5M Genomic DNA extraction kit, MagJET Genomic DNA kit (Thermo Fischer Scientific, Waltham, MA, USA), and HiGeneTM Genomic DNA Prep Kit (Biofact, Daejeon, Korea)), the purity and yield of the extracted gDNAs were determined based on the ratio of absorbance at wavelengths of 230, 260, and 280 nm, using a spectrophotometer (Nano-200, AllSheng, Hangzhou City, China).

### 2.9. Detection of Bacteria by PCR and qPCR

The primer used in this study was based on the coding sequence of the intimin adherence protein in the *eae* gene of *E. coli* O157:H7, one of the genetic markers for the serotype [25], with an amplicon size of 150 base pairs, the nucleotide sequence was (GGCGGATTAGACTTCGGCTA) for the forward primer and (CGTTTTGGCACTATTTGCCC) for the reverse primer. PCR reagents were used for conventional PCR, and the temperature was maintained using the MJ MINI thermocycler (Bio-RAD, Hercules, CA, USA). PCR products were separated based on size for 40 min at 100 V using a 2% agarose gel. LightCycler Nano (Roche, Basel, Switzerland) was used for qPCR, and the cycle threshold (Ct) was automatically determined. The same primers were used for both PCR and qPCR.

### 2.10. Statistical Data Analysis

Data representation is based on the mean ± standard deviation of three separate experiments. We used Student’s *t*-test to compare data under various conditions. Data was considered significant if P-value was less than 0.05.

## 3. Results and Discussion

### 3.1. Effect of Microchannel Geometry on Bacterial Capturing Efficiency

**Figure 3:**
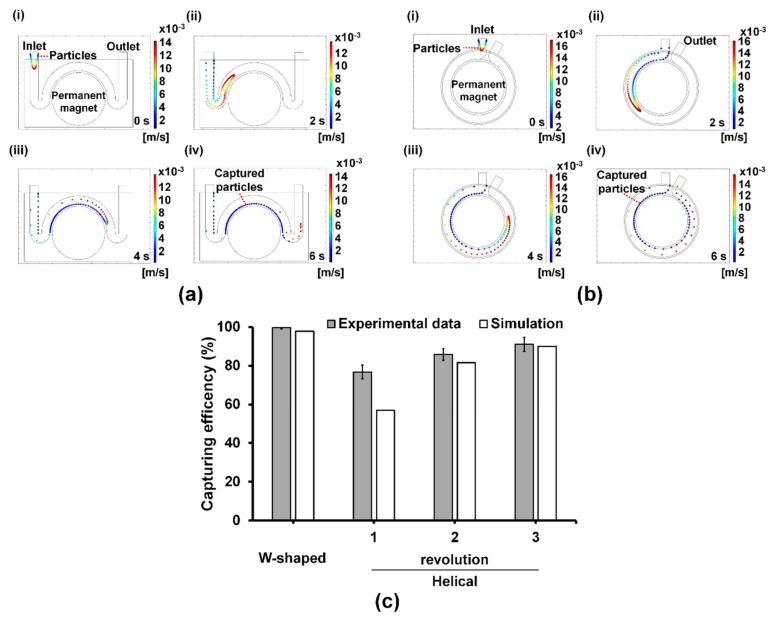
Comparison between simulation and experimental results. CFD simulation by COMSOL Multiphysics® software (ver. 5.1) in the W-shaped (**a**) and helical (**b**) microchannels (Figure S1). Each dot and its color represent a single bacterium-Ab-MNP complex and its velocity, respectively. In the simulation bacterium-Ab-MNP complexes (one hundred complexes) were injected into the inlet of both microchannels at 0 s at a flow rate of 2 mL/min, while a magnetic field was applied to the complexes from the center of the microchannel. Average magnetic field applied to the W-shaped and helical microchannels was 528 mT and 115 mT, respectively. (**c**) Simulation and experiment results of bacterial capturing efficiency for the W-shaped and helical microchannels. Bacterial capturing efficiency was estimated by counting the number of the complexes in the channel and outlet.

The bacterial capturing efficiency in the W-shaped microchannel was compared to that in the helical microchannel which was conventionally used. Our simulation results show the flow of bacterium-Ab-MNP complexes in W-shaped microchannel and helical microchannel (Figure 3a,b). Each dot and its color represent a single bacterium-Ab-MNP complex and its velocity, respectively. In W-shaped and helical microchannels, bacterium-Ab-MNP complexes were injected into the inlet at 0 s at a flow rate of 2 mL/min. At 2 s, bacterium-Ab-MNP complexes in the W-shaped microchannel increased momentarily due to the attraction of the magnet after passing through the first curve, but decreased in their speed at the beginning of the second curve (Figure 3a, ii), while those in the helical microchannel showed a linear velocity distribution and moved along the channel (Figure 3b, ii). At 4 s, bacterium-Ab-MNP complexes in the W-shaped microchannel were attached at higher concentrations to the left side of the second curve than in other regions due to the rate reduction occurred at 2 s (Figure 3a, iii), whereas those in the helical microchannel were attached along its inner wall of the channel, evenly spaced at a relatively low concentration, unlike in the case of W-shaped microchannel (Figure 3b, iii). At 6 s, most of the bacterium-Ab-MNP complexes in the W-type microchannel were attached to the channel wall and the remaining small amount of bacterial -Ab-MNP complexes were moved to the outlet (Figure 3a, iv). Bacterium-Ab-MNP complex in the helical microchannel were also attached to the channel wall, but a relatively large amounts of bacterium-Ab-MNP complexes were moved to the outlet compared to the W-type microchannel (Figure 3a, iv).

The simulation result was supported by the capturing efficiency calculation (Figure 3c). The results revealed that the capturing efficiency of the W-shaped microchannel was higher than the helical microchannel after three revolutions. Due to the tight curve in W-shaped microchannel before the wide curve at the lateral region of the permanent magnet, the, bacterium-Ab-MNP complexes in the flow were forced to sidewall at the beginning of the wide curve where the magnet was situated. As such, due to the pull on the bacterium-Ab-MNP complexes and the flow velocity along the wall, they decreased velocity. As a result, relatively more bacterium-Ab-MNP complexes were attracted by the magnetic force at the lateral region of the permanent magnet at the interconnection between the tight and wide curved channel section. Owing to this geometrical characteristic of the flow channel, the W-shaped microchannel exhibited better performance as a preconcentrator demonstrating a higher capturing efficiency when compared with the helical microchannel.

### 3.2. Comparison of Bacterial Capturing Efficiency at Different Flow Rates in the W-Shaped Microchannel

**Figure 4:**
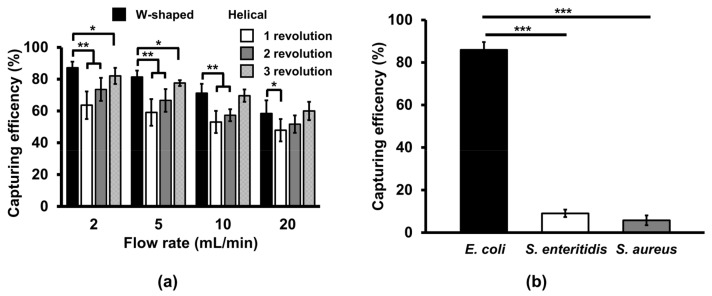
(**a**) Comparison of bacteria capturing efficiency in the W-shaped and helical microchannels at different revolutions and flow rates. (**b**) Cross-reactivity of Ab-MNPs with either *S. aureus* or *S. enteritidis* at 10^5^ CFU/mL in 2.5 mL of blood at the flow rate of 5 mL/min in the W-shaped microchannels. Student’s *t*-test, ***: *P* < 0.001. **: *P* < 0.01.*: *P* < 0.05. n = 3.

Bacterial capturing efficiency of both types of microchannels were tested by flowing the mixture of *E. coli* O157:H7 and MNPs conjugated with an antibody specific to the serotype in blood. At flow rates of 2, 5, 10, and 20 mL/min, the W-shaped microchannel had higher bacterial capturing efficiencies than the helical microchannel (1 to 3 revolutions) (Figure 4a). Increasing the number of revolutions for the helical microchannel have a little effect on the capturing efficiency. At 10 mL/min, the W-shaped microchannel still had higher bacterial capturing efficiencies than the helical microchannel with one and two revolutions. At 20 mL/min, the W-shaped microchannel had higher bacterial capturing efficiency than the helical microchannel with only one revolution. Overall, the capturing efficiencies in both types of microchannels tended to decrease as flow rates increased. These results show that the performance of the W-shaped microchannel is superior in capturing bacteria bound with Ab-MNPs than the helical microchannel. Because there is no significant difference in capturing efficiency between 2 mL/min and 5 mL/min in the W-shaped microchannel, 5 mL/min was chosen as the optimal flow rate for further experiments.

Bacterial capturing efficiency in the W-shaped microchannels (Figure 4a) was tested by flowing the mixture of *E. coli* O157:H7 and Ab-MNPs in blood. At different flow rates (2–20 mL/min), the W-shaped microchannel had the highest bacterial capturing efficiencies at 2 mL/min and the lowest capturing efficiency at 20 mL/min. Overall, the capturing efficiencies tended to decrease as flow rates increased. These results show that the performance of the W-shaped microchannel is superior in capturing bacteria at a flow rate of 2 mL/min and 5 mL/min. Because there is no significant difference in capturing efficiency between 2 mL/min and 5 mL/min in the W-shaped microchannel, 5 mL/min was chosen as the optimal flow rate for further experiments.

### 3.3. Cross-Reactivity of the Ab-MNPs

From 2.5 mL of blood, 86% of *E. coli* O157:H7 cells at 10^5^ CFU/mL was captured, while only 5% and 9% of *S. aureus* and *S. enteritidis* at 10^5^ CFU/mL were captured, respectively (Figure 4b). This shows that non-specific binding of Ab-MNPs to non-target microorganisms and adsorption of microorganisms onto the microchannel are negligible.

### 3.4. Improvement of Bacterial gDNA Purity by Adding a Conical Microchamber into the W-Shaped Microchannel

Blood samples spiked with *E. coli* O157:H7 at various concentrations (10^1^–10^6^ CFU/mL) were used as a template for PCR and qPCR. However, the target gene *eae* was not amplified under conditions (Figure S2). This is due to interference from PCR inhibitors such as hemoglobin [10,12], heparin [9], and lactoferrin [10] present in whole blood.

**Figure 5:**
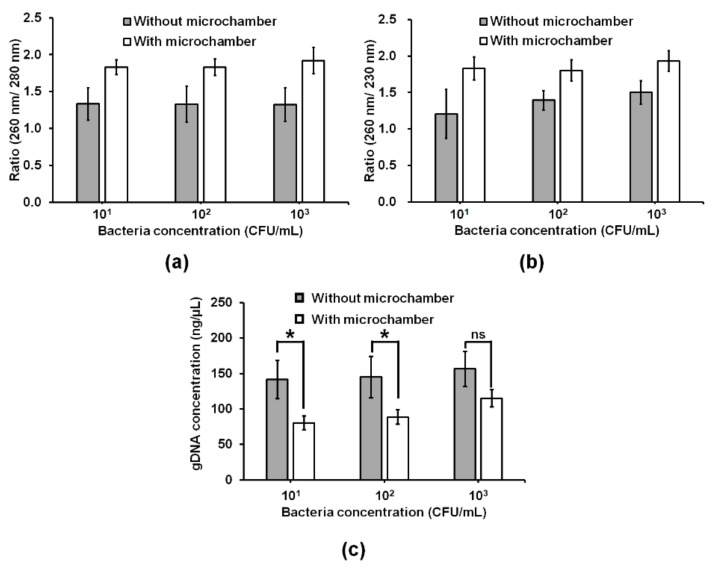
(**a**,**b**) Purity and (**c**) concentration graphs of bacterial gDNA obtained from 2.5 mL of blood spiked with *E. coli* O157:H7 at different concentrations (10^1^–10^3^ CFU/mL) using the W-shaped microchannels with and without a conical microchamber. Absorbances of protein, chemical and DNA are 280 nm, 230 nm and 260 nm, respectively. Student’s *t*-test. *: *P* < 0.05. n = 3.

To minimize biofouling of MSBs by blood [14], a conical microchamber for purification of bacterial gDNA was separated from the W-shaped microchannel (Figure 2a,b). In the microchamber, MSBs were mixed only with preconcentrated bacterial cells bound with Ab-MNPs by opening the valve between the microchamber and the W-shaped microchannel. The concentrations of gDNA obtained by the W-shaped microchannel without the conical microchamber at the different concentrations (10^1^–10^3^ CFU/mL) of *E. coli* O157:H7 in 2.5 mL of blood were higher than those obtained by the W-shaped microchannel with the conical microchamber (Figure 5c). However, the ratio of absorbance at 260 nm and 280 nm (A260/280) of bacterial gDNA purified by the W-shaped microchannel with and without the microchamber was approximately 1.8 and 1.3, respectively. Similarly, the ratio of absorbance at 260 nm to absorbance at 230 nm (A260/230) of the bacterial gDNA purified by the W-shaped microchannel with and without the microchamber was approximately 1.8 and 1.5 or lower, respectively. These results indicate that the bacterial gDNA obtained without the microchamber was not as pure and contaminated with protein (Figure 5a) or chemical contaminants (Figure 5b). These results show that separating the bacterial capturing microchannels and gDNA extraction microchambers improves gDNA purity. Taken together, it is suggested that the exposure of MSBs to blood should be minimized in order to protect the silica surface from being fouled by blood.

### 3.5. Improvement of Amplification of Bacterial gDNA by Adding a Conical Microchamber into the W-Shaped Microchannel

**Figure 6:**
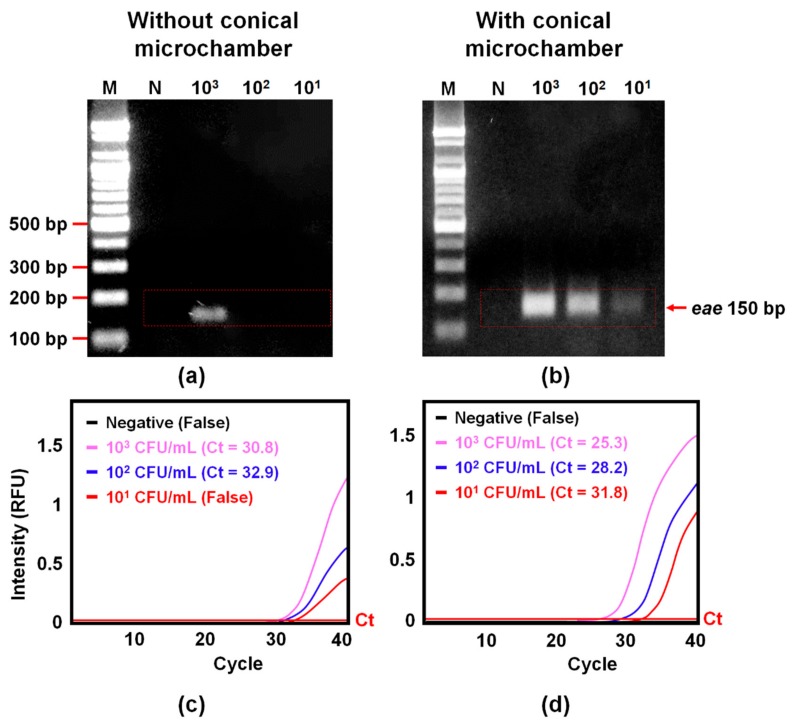
Confirmation of *E. coli* O157:H7 preconcentration and gDNA extraction efficiency using either PCR with gel electrophoresis (**a** and **b**) or qPCR (**c** and **d**) on the W-shaped microchannel with and without the conical microchamber. Blood samples (2.5 mL) containing *E. coli* O157:H7 at different concentrations (10^1^–10^3^ CFU/mL) were processed with the W-shaped microchannel either without the chamber (a and c) or with the chamber (b and d) before PCR and qPCR. Ct: cycle of threshold.

Quality of bacterial gDNA purified by the W-shaped microchannel with and without the microchamber was further assessed using PCR and qPCR. The sample preparation (*E. coli* O157:H7 preconcentration and gDNA purification) in the W-shaped microchannel improved the amplification performance of PCR to such a degree that 10^3^ CFU/mL (Figure 6a), while the one in the W-shaped microchannel with the microchamber improved the amplification performance of PCR to 10^1^ CFU/mL (Figure 6b). Similarly, the sample preparation in the W-shaped microchannel improved the amplification performance of qPCR to 10^2^ CFU/mL (Figure 6c), while the one in the W-shaped microchannel with the microchamber improved the amplification performance of qPCR to 10^1^ CFU/mL (Figure 6d). These results showed that the amplification of bacterial gDNA by PCR and qPCR was better improved by adding the microchamber into the W-shaped microchannel.

gDNA purification by the commercial kits improved the amplification performance of PCR and qPCR to such a degree that 10^4^ CFU/mL (Figure S3). There is an improvement of two orders of magnitude improvement when compared with samples prepared with commercial kits. The purity of bacterial gDNA purified by the kits (Table S1) was lower than that of bacterial gDNA obtained by the W-shaped microchannel with the microchamber (Figure 5a,b). By operating bacterial capturing and bacteria gDNA extraction in a separate place in the microfluidic device in a sequential manner, we can reduce contamination from chemicals and proteins. The small volume of the conical microchamber allows the use of a small amount of elution buffer, and the extracted gDNA sample has high purity and high concentration (Figure 5). The results suggest that our method lowers the LOD of molecular diagnostics for pathogens in blood by providing target bacterial gDNA at high purity and concentration.

## 4. Conclusions

In this study, we pioneered a 3DpmμFD consisting of a W-shaped microchannel and a conical microchamber to improve the LOD of molecular diagnostics in blood. The platform showed promising performance in improving LOD by removing inhibitory compounds and enriching the target bacteria at a high flow rate and reducing MSB contamination by separating the bacteria capturing microchannel and gDNA extraction microchamber. Our platform was able to effectively preconcentrate and purify a large volume of samples as compared with commercialized tube-based products. As a result, detection of as few as 10 *E. coli* O157:H7 CFU/mL in 2.5 mL of blood can be achieved by the combined use of PCR and qPCR. The platform includes several manual handling steps, which are cumbersome. It can be convenient through the automation of the handling steps. With this automation, it can be used to lower the LOD of molecular diagnostics for pathogens, including virus, in various types of samples, including blood and food, containing compounds inhibitory with gene amplification.

## Supplementary Materials

The following are available online at https://www.mdpi.com/1424-8220/20/4/1202/s1, Figure S1: The helical microchannels for magnetic preconcentration of bacteria of interest. Figure S2: Amplification of a target gene (*eae*) in *E. coli* O157:H7 in blood by PCR and quantitative PCR (qPCR) without any DNA purification process. Figure S3: Amplification of the target gene (eae) in *E. coli* O157:H7 in blood with the use of commercial DNA purification kits (MagListoTM 5M Genomic DNA extraction kit, MagJET Genomic DNA kit, and HiGeneTM Genomic DNA Prep Kit) by PCR and qPCR. Table S1: Concentration and purity of bacterial gDNA obtained by commercial DNA purification kits (MagListo^TM^ 5M Genomic DNA extraction kit, Bioneer Co. Daejeon, Korea; MagJET Genomic DNA kit, Thermo Fischer Scientific, Waltham, MA; HiGene^TM^ Genomic DNA Prep Kit, Biofact, Daejeon, Korea) at different concentrations of *E. coli* O157:H7 in 200 μL of blood.
