# Supplementary material for: 3D-Printed Modular Microfluidic Device Enabling Preconcentrating Bacteria and Purifying Bacterial DNA in Blood for Improving the Sensitivity of Molecular Diagnostics
Source: Sensors (Basel). 2020 Feb 21;20(4):1202. doi: 10.3390/s20041202 (PMC7070462; doi:10.3390/s20041202)
Supplement: Supplementary file 1 [file sensors-20-01202-s001.pdf]

sensors

MDPI

Article

3D-Printed Modular Microfluidic Device Enabling Preconcentrating Bacteria and Purifying Bacterial DNA in Blood for Improving the Sensitivity of Molecular Diagnostics

Abdurhaman Teyib Abafogi [1], Jaewon Kim [1], Jinyeop Lee [1], Merem Omer Mohammed [2], Danny van Noort [4,5,6], and Sungsu Park [1,2,3,*]

1 School of Mechanical Engineering, Sungkyunkwan University, Suwon 16419, Korea; ab18aa@gmail.com (A.T.A.); jaewon1394@gmail.com (J.K.); softmemsljy@naver.com (J.L.)
2 Department of Biomedical Engineering, Sungkyunkwan University, Suwon 16419, Korea; meremomer3@gmail.com (M.O.M.)
3 Biomedical Institute for Convergence at SKKU (BICS), Sungkyunkwan University, Suwon 16419, Korea
4 Division of Biotechnology, IFM, Linkoping University, Linkoping 58183, Sweden; drr.dvn@gmail.com
5 Chair of Micro Process Engineering and Technology (COMPETE), University of Ljubljana, 1000 Ljubljana, Slovenia
6 Centro de Investigación en Bioingeniería -BIO, Universidad de Ingenieria y Tecnologia—UTEC, Barranco 15036, Peru
* Correspondence: nanopark@skku.edu; Tel.: +82-31-290-7431; Fax: +82-31-290-5889

Received: 17 January 2020; Accepted: 20 February 2020; Published: 21 February 2020

Table S1. Concentration and purity of bacterial gDNA obtained by commercial DNA purification kits (MagListo™ 5M Genomic DNA extraction kit, Bioneer Co. Daejeon, Korea; MagJET Genomic DNA kit, Thermo Fischer Scientific, Waltham, MA, USA; HiGene™ Genomic DNA Prep Kit, Biofact, Daejeon, Korea) at different concentrations of *E. coli* O157:H7 in 200 μL of blood. The purity and yield of the extracted gDNA were determined based on the ratio of absorbance at wavelengths of 230, 260 and 280 nm, using a spectrophotometer (Nano-200, AllSheng, Hangzhou, China).

Commercial DNA Extraction Kit	Bacteria Concentration (CFU/mL)	gDNA Concentration (ng/μL)	Purity	
			260/280	260/230
MagListo™ 5M Genomic DNA extraction kit	10	12.68	1.71	1.99
	100	15.02	1.70	2.15
	1000	16.76	1.67	2.04
MagJET Genomic DNA kit	10	139.89	0.77	0.81
	100	203.02	0.98	0.87
	1000	189.12	1.49	2.60
HiGene™ Genomic DNA Prep Kit	10	100.32	1.11	1.25
	100	98.99	1.08	1.27
	1000	112.91	1.20	1.37

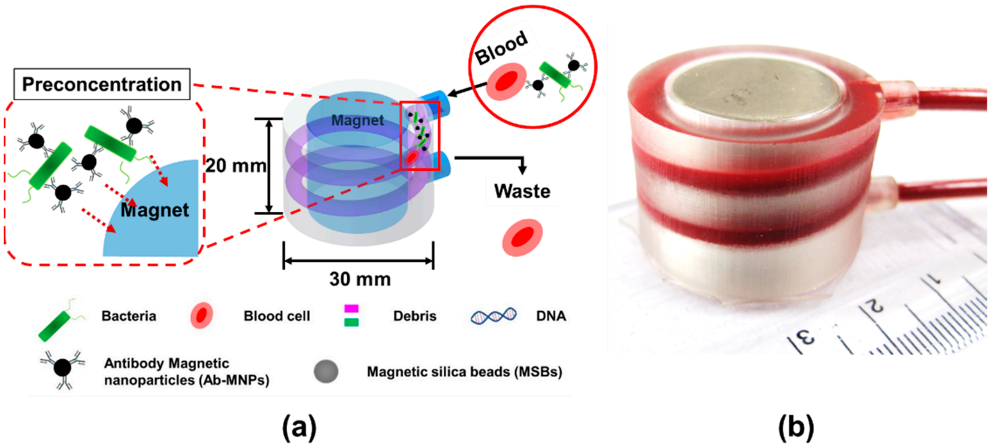

Figure S1. The helical microchannel for magnetic preconcentration of bacteria of interest. (**a**) A schematic with dimension of the helical microchannel. (**b**) Its photographic image.

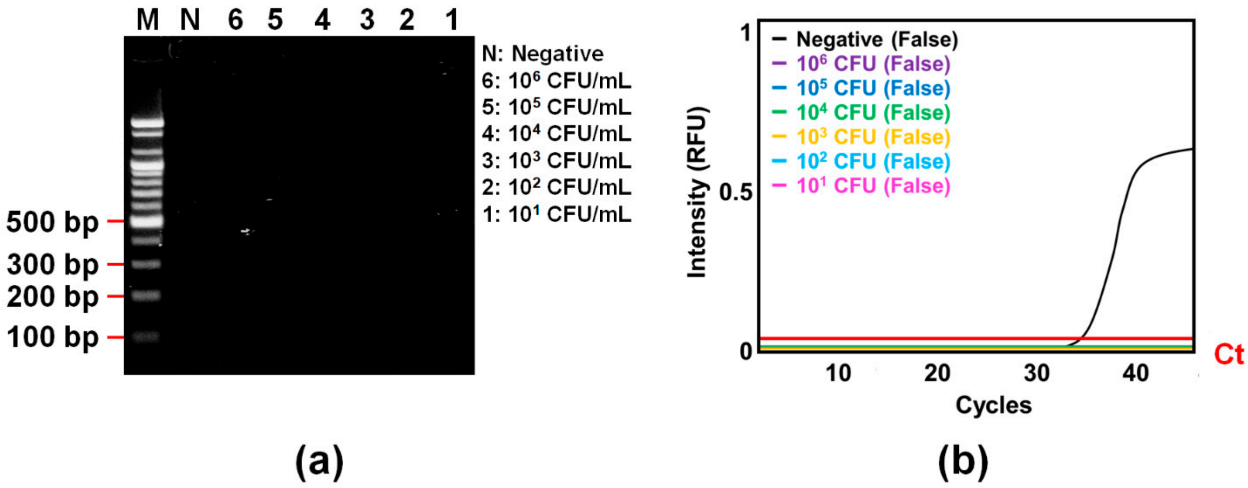

Figure S2. Amplification of a target gene (*eae*) in *E. coli* O157:H7 in blood by PCR and quantitative PCR (qPCR) without any DNA purification process. (**a**) PCR with gel electrophoresis and (**b**) qPCR of *eae* gene in *E. coli* O157:H7 at different concentrations (10–10^6 CFU/mL) from 2 µL of blood. Ct: cycle threshold. The primer used in this study was based on the coding sequence of the intimin adherence protein in the *eae* gene of *E. coli* O157:H7 with an amplicon size of 150 base pairs, the nucleotide sequence was (GGCGGATTAGACTTCGGCTA) for the forward primer and (CGTTTTGGCACTATTTGCCC) for the reverse primer. PCR reagents were used for conventional PCR, and the temperature was maintained using the MJ MINI thermocycler (Bio-RAD, Hercules, CA,). PCR products were separated based on size for 40 min at 100 V using a 2% agarose gel. Light Cycler Nano (Roche, Basel, Switzerland) was used for qPCR, and Ct was automatically determined. The same primers were used for both PCR and qPCR. Ct: cycle of threshold.

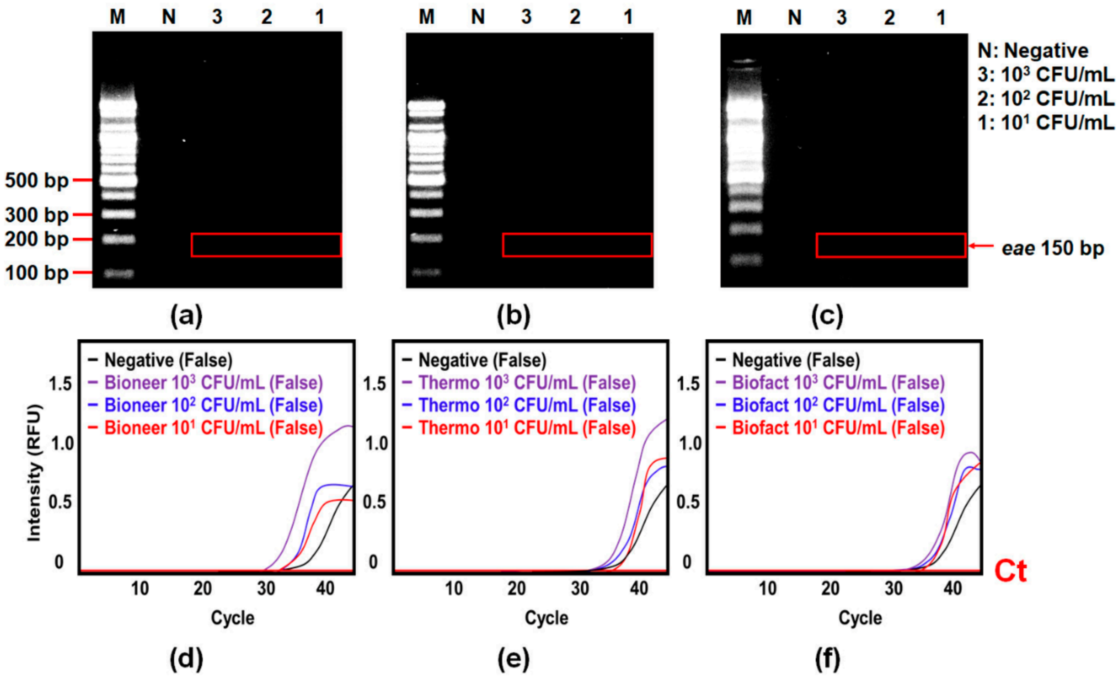

Figure S3. Amplification of the target gene in *E. coli* O157:H7 in blood with the use of commercial DNA purification kits (MagListo™ 5M Genomic DNA extraction kit, Bioneer Co. Daejeon, Korea; MagJET Genomic DNA kit, Thermo Fisher Scientific, Waltham, MA; HiGene™ Genomic DNA Prep Kit [Magnetic Bead] for cultured cell, Biofact, Daejeon, Korea) by PCR and qPCR. (**a**–**c**) PCR with gel electrophoresis and (**d**–**f**) qPCR of *eae* gene in *E. coli* O157:H7 at different concentrations (10–10^3 CFU/mL) from 200 μL of blood purified by either Bioneer DNA extraction kits or Thermo Fischer Scientific DNA extraction kits (b,e) or Biofact DNA extraction kit (d–f).

© 2020 by the authors. Licensee MDPI, Basel, Switzerland. This article is an open access article distributed under the terms and conditions of the Creative Commons Attribution (CC BY) license (http://creativecommons.org/licenses/by/4.0/).
